# The Crooked Nose—Surgical Algorithm in Post-Traumatic Patient—Evaluation of Surgical Sequence

**DOI:** 10.3390/jcm14010087

**Published:** 2024-12-27

**Authors:** Marcin Jadczak, Sandra Krzywdzińska, Paweł Rozbicki, Dariusz Jurkiewicz

**Affiliations:** Department of Otolaryngology with Division of Cranio-Maxillo-Facial Surgery, Military Institute of Medicine—National Research Institute, 04-141 Warsaw, Poland; mjadczak@wim.mil.pl (M.J.); prozbicki@wim.mil.pl (P.R.); djurkiewicz@wim.mil.pl (D.J.)

**Keywords:** rhinoseptoplasty, nasal obstruction, crooked nose, quality of life, nasal trauma

## Abstract

**Background:** A crooked nose is a challenge for a surgeon performing rhinoplasty. When performed correctly, rhinoseptoplasty aligns the nasal framework, restores nasal patency, and achieves facial symmetry. The key to this procedure is to dissect all the structures of the nasal framework, mobilize, reposition, and stabilize them. **Aim:** This study aims to discuss the quality of life after the rhinoseptoplasty and principles of treating a post-traumatic crooked nose with a view to improving the predictability and reliability of rhinoplasty procedures involving this challenging problem. **Methods:** The study compared the results of the Rhinoplasty Outcome Evaluation (ROE) and the Standardized Cosmesis and Health Nasal Outcomes Survey (SCHNOS) through statistical analysis. **Results:** Considering the structural deformities that lead to a crooked nose, the open approach seems to be preferred during a rhinoseptoplasty of a post-traumatic, crooked nose. When reconstructing the nasal septum, it is always necessary to leave the required amount of cartilage to provide nasal support and to secure the septum to the nasal spine. Osteotomies are important for fixing a crooked nose. The preoperative values for ROE were significantly lower before surgery than after surgery (8.7 vs. 20.2), while for SCHNOS, the postoperative values were statistically significantly lower compared to the preoperative values (46.0 vs. 9.1). **Conclusions:** Properly planned and performed surgery improves the functional and aesthetic outcomes in patients after rhinoseptoplasty.

## 1. Introduction

Fixing a crooked nose is a major reason for patients to qualify for a rhinoseptoplasty. A curved shape results from the deviation of the nose components from the midline of the face; the nose may have asymmetries and irregularities that disrupt both its appearance and patency. For this reason, patients with a crooked nose complain not only of aesthetic but also functional problems [1,2]. A crooked nose can be congenital or acquired as a result of trauma or unsuccessful surgery. Fixing a crooked nose poses a considerable challenge for the surgeon, as the deformity of the external nose is usually reflected by its internal structure. A crooked nose results from the asymmetry of the cartilage and bones that make up the nose. The nasal septum plays a particularly important role. According to the literature, a deviated septum is the most commonly described pathology causing a crooked nose, giving rise to a popular phrase by Maurice Cottle: “As the septum goes, so goes the nose” [3,4]. In addition, factors such as unevenly thickened subcutaneous tissue, ligaments, osteochondral attachments, including attachments between the bony pyramid, lateral cartilage, alar cartilage, and nasal septum, or contractures secondary to scarring after trauma or surgery, should not be overlooked [5,6]. Cultural aspects are also important to consider. For instance, in individuals of Asian, African, and Middle Eastern descent, nasal anatomy often differs in terms of nasal dorsum, tip projection, and skin thickness. Asian noses may feature a flatter nasal bridge, while African or African American noses may have broader, more prominent nasal bases with thicker skin. These variations not only affect the aesthetic outcome of post-traumatic rhinoplasty but can also impact functional considerations, such as airflow and airway obstruction [7].

A crooked nose significantly affects a patient’s self-esteem and quality of life. The aim of this study is to develop and present an algorithm that assists surgeons in the operation of a crooked nose and to evaluate the improvement in quality of life for patients after the surgical procedure using validated questionnaires. As far as we know, this study is the first report to test the outcomes of a surgical algorithm for traumatic nasal deformities, comparing preoperative and postoperative scores by using a subjective measurement method: the Rhinoplasty Outcome Evaluation (ROE) and the Standardized Cosmesis and Health Nasal Outcomes Survey (SCHNOS) questionnaires.

## 2. Materials and Methods

The study involved 89 patients (63 females and 26 males) at the mean age of 35.4 ± 10.2 years, range 18–57 years, admitted to the Department of Otolaryngology with the Division of Cranio-Maxillo-Facial Surgery at the Military Institute of Medicine—National Research Institute in Warsaw and qualified for primary rhinoseptoplasty surgery due to post-traumatic deformation of the external nose and disturbances in the patency of the nasal cavities.

The inclusion criteria were age over 18, a negative history of paranasal sinus disorders, history of nasal trauma, significant deviation of the nasal pyramid, and obstruction of one of the nasal cavities.

The exclusion criteria were no history of trauma, patients who had previous corrective rhinoplasty, cleft nose deformity, age under 18, diseases of the paranasal sinuses, acute nasal trauma or nasal bone fracture up to 6 months before surgery, additional concomitant procedures (e.g., functional endoscopic sinus surgery, blepharoplasty), a history of depression, body dysmorphic disorder (BDD), lack of consent for photographic documentation, and lack of consent to participate in the study.

Patients received feedback verbally and in writing. Conscious agreement was obtained from all individual study participants. The research was approved by The Ethical Committee of the Military Chamber of Physicians, KB/5/24, KB/24/24. The patients consented to the publication of their images.

Patients completed questionnaires before the procedure, one month and six months after the operation. The form included the Rhinoplasty Outcome Evaluation (ROE) and the Standardized Cosmesis and Health Nasal Outcomes Survey (SCHNOS) questionnaires.

The ROE is a simple, self-reported questionnaire designed to evaluate patient satisfaction in the rhinoplasty cases. This tool consists of a few key questions that assess both aesthetic and functional aspects of the nose. It also shows the impact of rhinoplasty on the patient’s quality of life [8].

The SCHNOS is a questionnaire that combines both cosmetic and functional assessments of nasal surgery outcomes. SCHNOS includes specific questions that address two main areas: cosmetic (SCHNOS-C) and obstructive (SCHNOS-O) domains. By addressing both appearance and function, SCHNOS provides a more comprehensive overview of the patient’s experience post-surgery [9].

All patients underwent a detailed physical examination. Photographic documentation consisting of frontal, basal, lateral, and oblique views was made during each visit. During the study, 9 patients (1 man and 8 women) were excluded due to the inaccurate completion of the ROE and/or SCHNOS questionnaires.

The data were statistically analyzed.

In order to standardize the surgical results, an algorithm of surgical procedures was developed when caring for a patient with a crooked nose (Table 1).

### 2.1. Preoperative Planning and Preparation

The preoperative consultation is a fundamental aspect of surgical preparation, serving as a platform to understand the patient’s expectations and concerns regarding the procedure. A crucial component of the preoperative consultation is to identify nasal deformities and disproportions as well as evaluate the realistic outcomes that can be achieved through surgery. It is important to have an open discussion with the patient about their expectations to ensure a mutual understanding of the attainable surgical results.

Patients should be informed that in cases of significant nasal deviation or severe aesthetic deformity, a “perfect” result may not be feasible, regardless of the surgeon’s expertise. In the case of a severely crooked nose, creating a perfectly straight and symmetrical outcome often proves to be an unattainable task. Defining and carefully discussing the patient’s expectations before the surgery is a crucial step in minimizing the risk of postoperative dissatisfaction [3,6,10].

**Figure 1:**
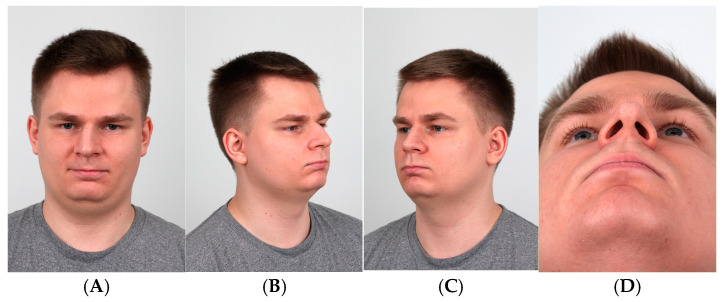
(**A**–**D**) Photographs of the views.

A comprehensive clinical analysis assessing facial proportions, together with the identification of any nasal abnormalities and structural deformities, is a key factor to achieve optimal outcomes in rhinoseptoplasty. The patient examination should consist of an external and internal evaluation of the nose, with particular attention to nasal cavity patency. The external examination should include a multi-angle assessment of the nose, involving frontal, lateral, oblique, basal, and superior/top views of the nose (Figure 1A–D) [2,6]. During the consultation, photographic documentation should be performed. Careful assessment of the photographs can uncover subtle deformities that might be missed during the physical examination. Moreover, reviewing the photographs with the patient is a helpful communication tool allowing for a clear presentation of the existing issues, an explanation of the surgical plan, and the establishment of a reference point for evaluating postoperative outcomes [11,12,13].

Particular emphasis is placed on identifying the abnormalities that contribute to a crooked nose, i.e., bone asymmetries and irregularities of any type, the shape of the dorsum, the tip of the nose, and the nostrils, as well as the deviation of these structures from the midline of the face.

The frontal view enables the assessment of nasal alignment in relation to overall facial asymmetry (Figure 1A). In certain cases, nasal asymmetries may be secondary to underlying facial asymmetries, limiting the potential for rhinoseptoplasty to fully correct these imbalances.

Oblique views are valuable for evaluating the width of the different parts of the nasal pyramid, identifying asymmetries, and detecting irregularities along the nasal dorsum (Figure 1B,C).

The basal view is crucial for assessing deviations of the septum in the caudal part, the position of the nasal tip nostril asymmetry (Figure 1D) [11,12,13].

### 2.2. Procedure Algorithm for a Crooked Nose

During surgery, it is beneficial to follow the algorithm, restoring symmetry to the various components of the nose, gradually improving the anatomical relationships within this complex structure. It is essential to address both the internal and external elements of the nasal architecture to achieve a satisfactory final outcome, as neglecting either aspect may compromise the overall success of the procedure.

#### 2.2.1. Open Approach

When performing rhinoseptoplasty in patients with a deviated nose, the open technique is generally preferred [2,3,6,10,12,13]. Closed rhinoseptoplasty can be unpredictable, especially during reoperation or after trauma where the original nasal anatomy has been disrupted. The presence of subcutaneous scarring or thickening of soft tissues often complicates the assessment of nasal structures, leading to the potential application of inadequate surgical techniques. The open approach provides enhanced visualization of nasal structures, allowing for thorough evaluation and real-time control during the procedure. This technique is particularly advantageous for releasing the adhesions between the soft tissue, cartilage, and bony components, which significantly contribute to external deformities—an especially crucial factor in revision or post-traumatic rhinoplasty [10]. Often, only after the individual components have been accurately exposed can the anatomical structures be fully identified. Furthermore, the open approach enables the surgeon to perform precise intraoperative assessments of the surgical techniques used and their impact on nasal symmetry [3,6,10].

#### 2.2.2. Septum Management

Reconstruction of the nasal septum, following the principle “as the septum goes, so goes the nose”, is a key component of rhinoseptoplasty [4]. Improperly treated nasal septum deviations are the main cause of unsuccessful rhinoplasty results [14]^.^

The procedure involves restoring the alignment of the nasal septum to the midline while ensuring that the remaining portion of the septum in the anterior segment maintains sufficient strength to support the nasal structure. The surgeon should aim to preserve as much of the cartilage as possible, removing only the deviated sections or harvesting the material required for grafts. To prevent the postoperative collapse of the nose, at least 12–15 mm of quadrangular cartilage should always be retained in the K-area, without detaching the cartilage from the ethmoid bone [14,15].

First, the (mucoperichondrial and mucoperiosteal) flaps should be lifted along the entire length of the nasal septum up to the anterior nasal spine. In cases of a deviated nose, the septum should be visualized bilaterally. With unilateral elevation of the mucosa and the perichondrium, asymmetrical forces may be created during the healing process, potentially leading to renewed septal deviation and, consequently, the reformation of the nasal pyramid deformity [14,15]. In the authors’ opinion, the open approach allows for a more precise assessment of the internal forces influencing nasal deviation.

**Figure 2:**
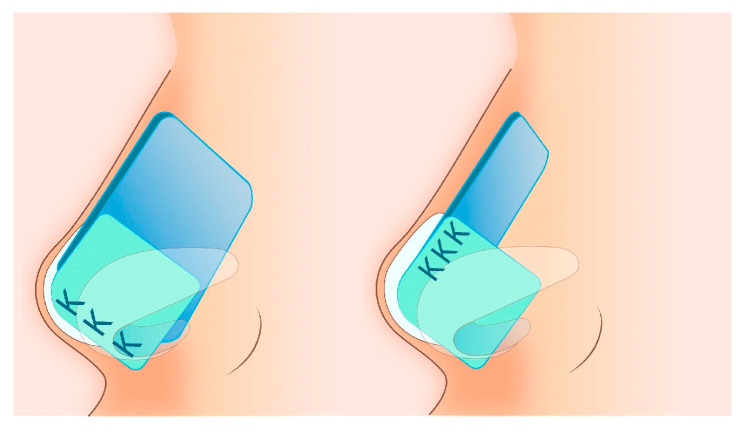
Splinting grafts: the figure shows the method of suture placement.

Subsequently, the quadrangular cartilage should be carefully separated from the maxillary bone and the nasal spine. An additional posterior chondrotomy allows for displacement and repositioning of the quadrangular cartilage along the midline. Importantly, leaving at least 14 mm of connected quadrangular cartilage with the perpendicular plate of the ethmoid bone beneath the nasal dorsum prevents the nasal pyramid from collapsing [14,15]. In cases of caudal subluxation of the quadrangular cartilage, a vertical septal excess near the anterior nasal spine can often be observed. Unilateral elimination of part of the nasal spine and removal of the excess ventral part of the septum allows for realignment along the midline. By mobilizing and fixing the septal cartilage at the nasal spine, both septal straightening and enhanced tip support, leading to elevation of the nasal tip, can be achieved. Additionally, straightening of a curved septum may involve scarification of the quadrangular cartilage on the concave side of the curvature, with the application of straightening sutures, splinting grafts, or suturing through the perforated perpendicular plate of the ethmoid bone to provide additional support (Figure 2) [6,10,12,14,15,16,17].

For angular deformities, wedge excision in combination with splinting grafts can achieve satisfactory outcomes. It should be borne in mind that the above-mentioned techniques may require more cartilage than is available from the septum, necessitating additional graft material from the patient’s ear or rib. A lack of adequate preoperative planning and insufficient cartilage may pose significant intraoperative challenges, particularly if consent for additional cartilage harvesting has not been obtained [16,17].

#### 2.2.3. Nasal Conchae Reduction

The size and structure of the nasal conchae must also be assessed during surgery. Many times, a massive concha bullosa prevents the straightening of the nasal septum. When this happens, its lateral wall must be removed during the procedure, resulting in a partial reduction of the middle nasal concha. In the case of the inferior nasal conchae, it is sometimes necessary to perform a lateralization (preferred by the authors) or another form of conchoplasty to obtain adequate space for breathing [5,16].

#### 2.2.4. Osteotomies and Osteoplasty

In traumatic cases, the surgeon encounters significant nasal bone deformities, and osteotomies are a tool that enables the correction of such deformities [18]. Osteotomies must be performed with caution, as they can cause, among other complications, temporary impairment of the sense of smell and damage to the tear ducts [2].

The primary purpose of osteotomies is to close, narrow, and correct curvatures of the nasal pyramid [13,16]. The bony pyramid of the nose can be conceptualized as an asymmetrical triangle; the longer bony wall should be shortened or made to appear short during osteotomy. This can be achieved by removing the nasal hump or reducing the height of the nasal bone on the longer side. Additionally, a strip of bone at the base of the nasal pyramid can be removed to create a space for repositioning the pyramid. Power instruments such as a piezo knife are ideal for this purpose, though the procedure can also be performed with surgical saws as well as osteotomes or Luer forceps [17,19,20].

Asymmetric osteotomies can also be considered. On the longer side of the nasal pyramid, an intermediate or an intermediate and lateral osteotomy can be performed, while on the shorter side, only a lateral osteotomy can be necessary. This technique allows for a measurable reduction in length and restoration of nasal symmetry [15,20].

#### 2.2.5. Middle Third of the Nose

**Figure 3:**
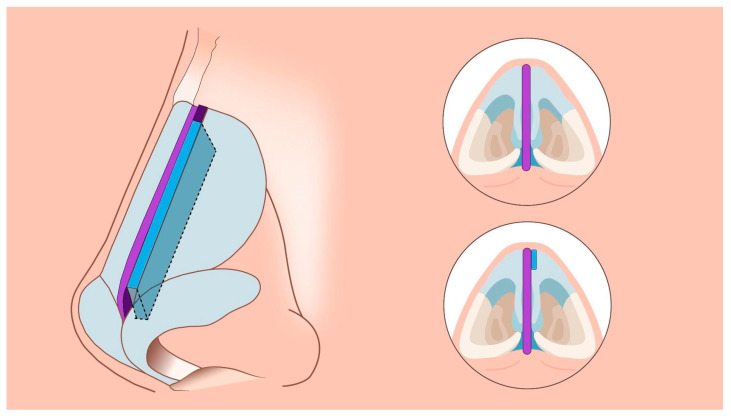
Fragment of cartilage, usually obtained from the nasal septum or, if there is insufficient material, from the auricular cartilage or rib cartilage, is placed between the nasal septal cartilage and the upper lateral cartilage of the nose to fill the collapse on the nasal dorsum and restore dorsum.

**Figure 4:**
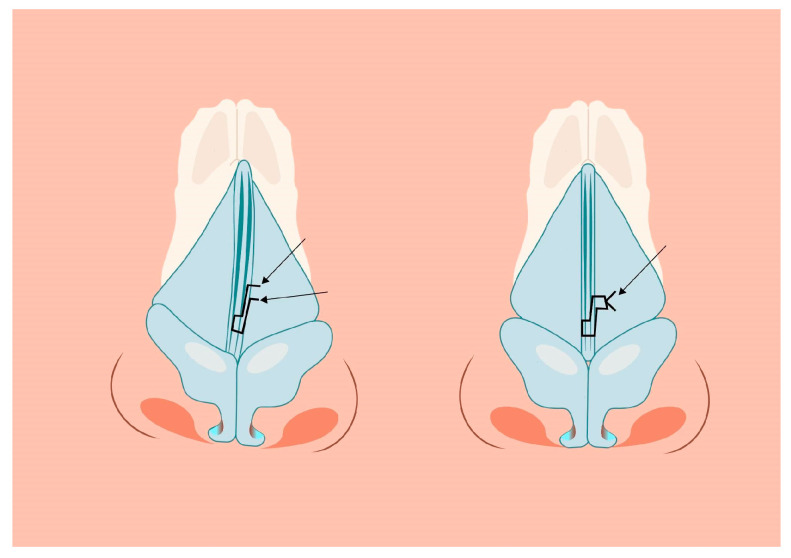
Clocking sutures reposition the caudal end of the septum aligning it with the midline.

Assuming successful straightening of the nasal septum during septoplasty, residual asymmetry or distortion of the nasal dorsum may still be present. The simplest way to restore nasal symmetry is the use of spreader grafts (Figure 3) [5,10,12,14,15]. After separation of the quadrangular cartilage, asymmetrical clocking sutures can be applied to reposition the caudal end of the septum, aligning it with the midline (Figure 4).

#### 2.2.6. Nasal Tip

**Figure 5:**
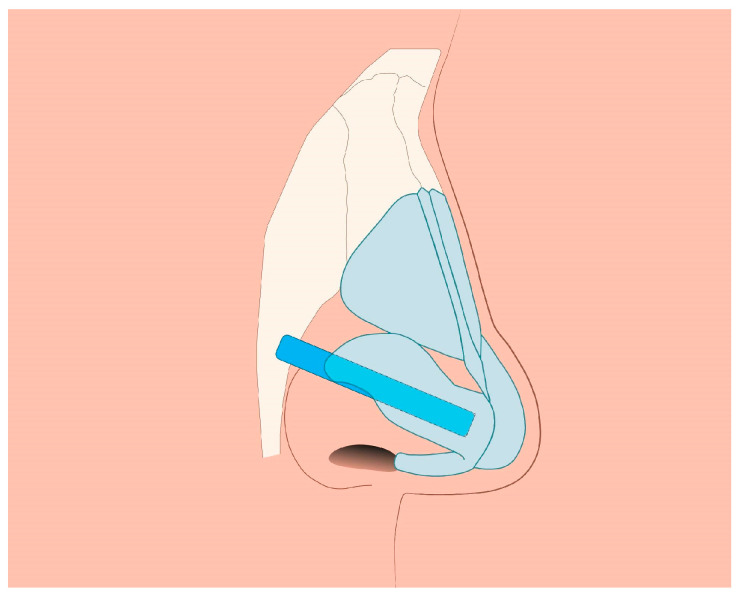
Lateral crural strut graft (LCSG).

Another crucial aspect is the restoration of the nasal tip by using sutures to refine the tip shape. At this stage, the knowledge, skill, and experience of the operator play a key role during the procedure. First, it is necessary to determine the anatomical abnormalities causing tip asymmetry. Amorphousness may result from the different shape and stiffness of the lateral crura and the asymmetric position of the dome of alar cartilages in relation to each other. The domes may lie at different levels, both in the anteroposterior direction as well as upwards and downwards relative to each other. They can also have different shapes. The lateral crura may have different rotation in the longitudinal axis and be located more or less cephalically asymmetrical compared to the contralateral side. Once the problem has been assessed, surgical techniques should be implemented to restore symmetry to the structure of the nasal tip complex. The following can be used to improve the shape of the lateral crus: cartilage scarification, cephalic trim, lateral crural strut graft (LCSG), asymmetrical lateral crus steal (ALCS), alar batten graft, and 7X suture (Figure 5) [21,22,23,24].

#### 2.2.7. Final Camouflage

The role of soft tissues is critical in rhinoseptoplasty. Dorsal irregularities that disrupt the symmetry of the nose may result from irregularities in the skin or excess thickened subcutaneous tissue in the supratip area. In such cases, slight trimming of the soft tissues of the nose (subcutaneous tissue, SMAS) or the placement of compressed cartilage on the dorsum in place of the deformity can significantly enhance symmetry. Any additional materials placed on the dorsum of the nose must be carefully arranged and securely fixed to adjacent structures. Inadequate or unstable arrangement can result in displacement of the grafts in the postoperative period and the development of unevenness and asymmetry—sometimes months after the procedure has been performed [2,3,25].

#### 2.2.8. Dressing

In order to stabilize the nose after surgery, it is important to place an external nose cast and a stabilizing plate on the nasal septum [14]. In the authors’ practice, a thermoplastic plate is most commonly used on the nasal dorsum. A tamponade is inserted into the nasal cavity and removed the day after surgery. The nasal septal plates and external dressing, along with the skin sutures, are removed on the seventh day postoperatively [25].

## 3. Statistical Analysis

Data obtained during the study were statistically analyzed with the use of software Statistica 13.1. Normal distribution in each group of variables was checked by the Kolmogorov–Smirnov test, where a *p*-value > 0.05 confirmed no statistical differences with normal distribution. Subsequently, to check the significance in groups before and after surgery, we performed non-parametric analysis, conducting the Mann–Whitney U test.

## 4. Results

In the study group, the following average values were obtained in the questionnaires analyzed (Table 2).

**Figure 6:**
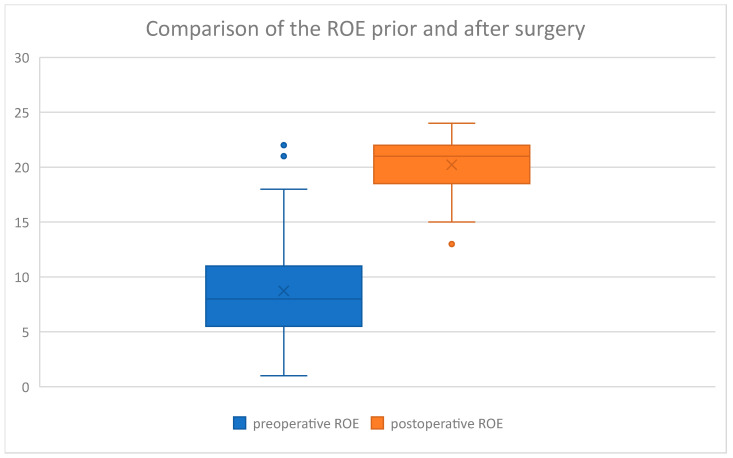
ROE preoperative vs. ROE postoperative.

**Figure 7:**
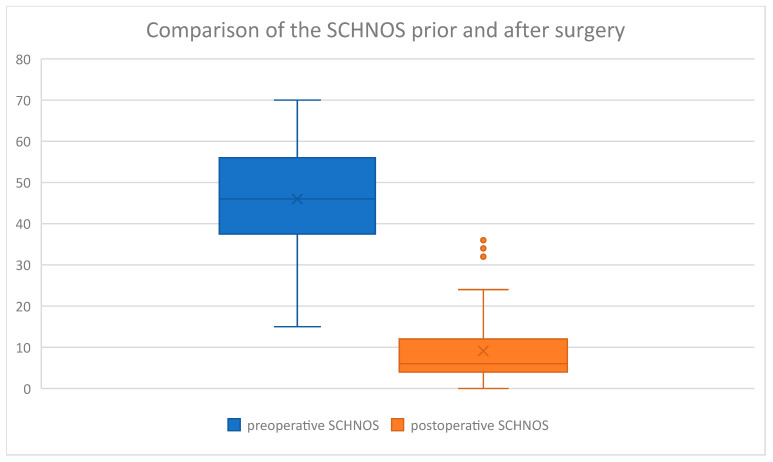
SCHNOS preoperative vs. postoperative.

**Figure 8:**
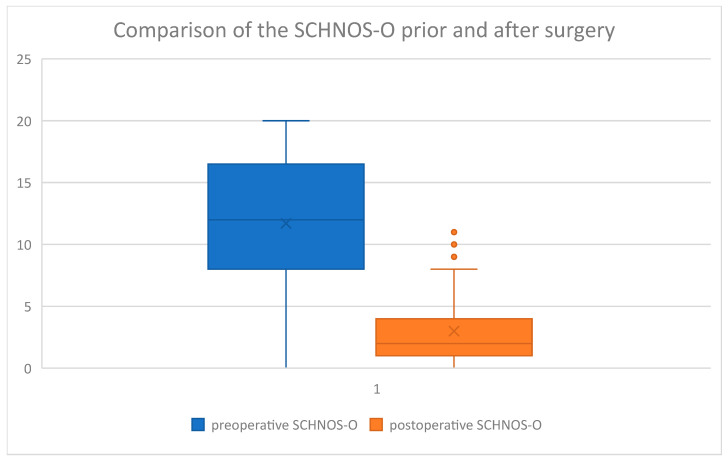
SCHNOS-O preoperative vs. postoperative.

**Figure 9:**
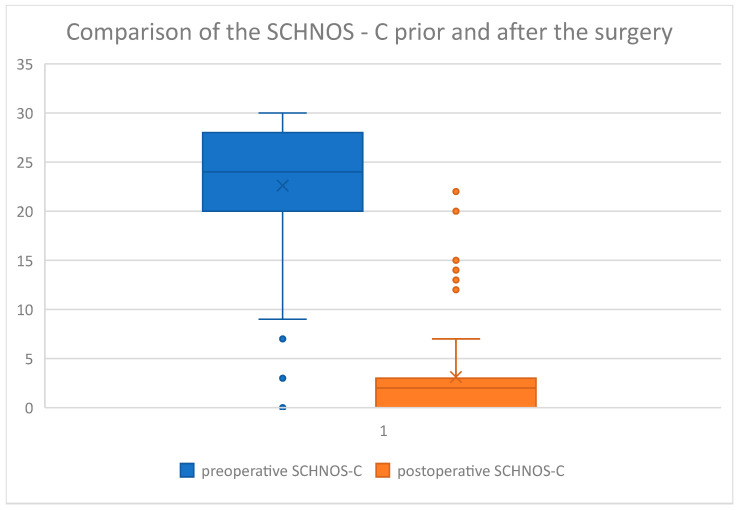
SCHNOS-C preoperative and postoperative.

The questionnaire results were then displayed on box charts (Figure 6, Figure 7, Figure 8 and Figure 9):

On the above-mentioned (Figure 6) visual increase in ROE score after surgery was observed in the examined group. Coterminously, rhinoplasty performed in our group of patients caused a decrease in SCHNOS (Figure 7), SCHNOS-O (Figure 8), and SCHNOS-C (Figure 9).

Subsequently, a Kolmogorov–Smirnov test was performed on the study group to assess the presence of a normal distribution (Table 3). The normal distribution was confirmed in ROE preoperative and SCHNOS preoperative, whereas the hypothesis of normality was rejected for ROE postoperative and SCHNOS postoperative.

Next, the significance of differences between the scores before and after the surgery was assessed using the Mann–Whitney U test (Table 4). This analysis revealed statistically significant differences between the scores before and after the rhinoseptoplasty. Considering the ROE, the postoperative scores were significantly higher (8.7 vs. 20.2), and for the SCHNOS, they were statistically significantly lower (46.0 vs. 9.1). SCHNOS-O and SCHNOS-C parameters were also analyzed, showing a statistically significant (*p* < 0.001) decrease in scores, translating into better postoperative outcomes.

### Visual Results

**Figure 10:**
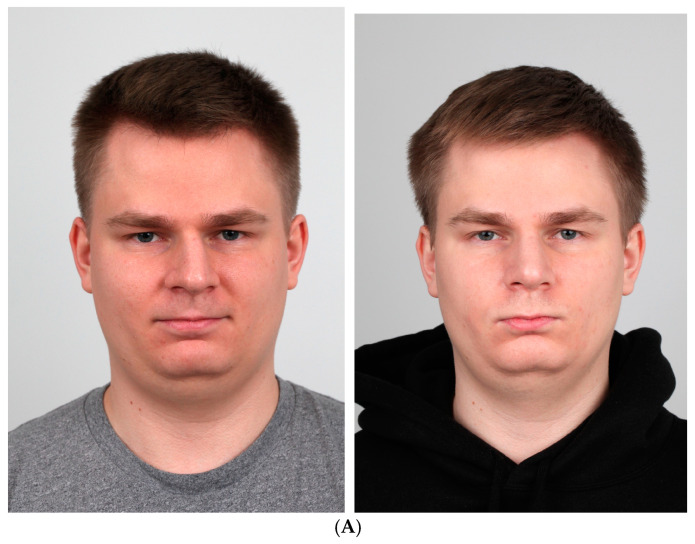

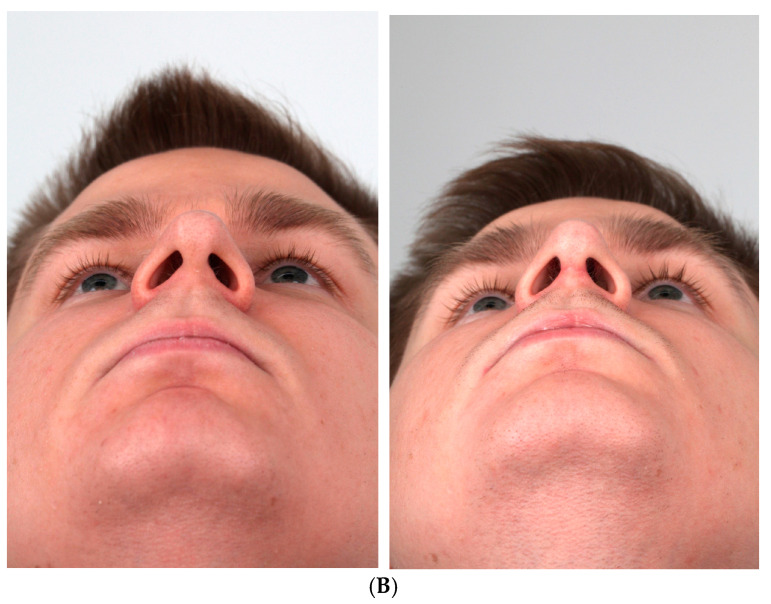
(**A**) pre- and postoperative images of the patient. Frontal view. (**B**) pre- and postoperative images of the patient. The basal view.

When performed correctly, the procedure produces functional as well as visual results. The figures show a pre- and postoperative comparison (Figure 1A,D and Figure 10A,B), a significant improvement in the symmetry and patency of the nose.

Immediately after the procedure, patients experienced bruising and swelling around the nose and eye, which gradually decreased over time. In two patients, postoperative wound infection occurred and was successfully treated with antibiotic therapy. There were no iatrogenic complications, including collapse of the central vault.

## 5. Discussion

The literature describes and compares many surgical techniques for correcting a crooked nose, depending on the cause, degree of deviation, and uniqueness of the patient’s anatomy. Correcting a crooked nose with long-term functional and aesthetic success remains the main challenge for the surgeon. This type of rhinoplasty, especially in post-traumatic cases where the natural anatomy and physiology of the nose have been altered, is often considered one of the most challenging procedures in facial surgery due to the asymmetry present and the structural abnormalities that a crooked nose causes.

In the above study, diseases of the paranasal sinuses, a history of depression, and a diagnosis of BDD were the exclusion criteria. Sinus disease can have structural and functional implications, possibly affecting the effectiveness of preoperative assessment and thus surgical outcomes. Similarly, people suffering from depression may have different expectations or psychological reactions to surgical outcomes, which may affect both subjective satisfaction and the reliability of postoperative assessment. BDD is characterized by obsessive and constant apprehension associated with the conviction that one’s appearance or physique is unattractive. In terms of facial plastic surgery, BDD poses unique challenges for both patients and surgeons, as it often leads to unrealistic and unattainable expectations, with a concomitant risk of dissatisfaction with surgical outcomes, regardless of the objective success of the procedure. Sarwer et al. [26] showed that patients with BDD are often dissatisfied with the surgery outcomes due to a distorted self-image, which is rooted in psychological factors rather than reality. Furthermore, it is believed that patients with BDD not only do not benefit from surgery or aesthetic medicine but often, after treatment, an exacerbation of BDD symptoms and an increase in the obsessive thoughts and actions in an individual are observed in this group [27,28]. For this reason, authors such as C. E. Crerand and M. Radman et al. suggested including a mental health assessment during the preoperative consultation [29]. Using the exclusion criteria described, the authors wanted to ensure the relevance of the study results and their applicability to a healthy population without confounding factors.

The differences found in patients with and without a history of previous nasal trauma are worth highlighting. In non-traumatic crooked nose cases, the nasal axis tends to deviate towards the smaller part of the face, which is also accompanied by a chin tilt [30]. Some authors explain this through facial growth patterns [31]. Although the controversy surrounding the reciprocal influence of nasal growth and the jaw complex continues today, many studies indicate that a crooked nose often accompanies other facial deformities, such as asymmetry and a lack of proportion on both sides of the face. Many authors have suggested that part of the cartilage of the nasal septum and nasal resistance may influence the growth of the jaw and the development of the nasomaxillary complex in early childhood [32,33]. Research suggests that the underlying cause of a non-traumatic crooked nose may be a genetic problem and abnormalities in the growth centers. These cause slower growth on one side of the nose, resulting in its deviation towards the slower-growing side of the face [34]. When correcting a crooked nose with concomitant facial asymmetry, the surgeon should aim to achieve symmetry of the nose by locating it on a line running from the center of the glabella to the center of the Cupid’s bow. This maneuver can reduce the apparent facial asymmetry, resulting in greater patient satisfaction [34]. However, this does not mean that the placement of the nose will be straight, but it will allow the facial symmetry to be visibly improved.

Rhinoseptoplasty of a crooked post-traumatic nose poses additional challenges for the surgeon. It must be well thought out and planned to achieve satisfactory functional and aesthetic outcomes. After trauma, deformed nasal structures lead to an irregular nose shape. The displacement of both the nasal bones and the nasal septum disturbs the facial appearance and causes breathing difficulties. Moreover, patients after nasal trauma often expect the surgeon to achieve a pre-injury effect or even a more satisfactory outcome. Remembering their nasal function and pre-trauma appearance, they often have higher expectations than patients born with a crooked nose, not a traumatic one [35]. Such patients are also likely to experience heightened emotional investment in the outcome, as the surgery addresses both aesthetic and psychological aspects tied to the injury [35,36]. Preoperative analysis, which includes a thorough history taking and physical examination, as well as planning that takes into account the surgeon’s ability to perform the various stages of the procedure accurately, is essential. An important and often emphasized aspect of facial plastic surgery is clear and complete communication with the patient. Surveys such as ROE, SCHNOS, photographic documentation, and visualizations made before the procedure and discussed with the patient appear helpful.

Several proposed algorithms for correcting a crooked nose are available in the literature [2,18,37,38,39,40]. Most did not evaluate postoperative outcomes. The algorithm described by the authors is intended to facilitate the surgical management of difficult cases, especially for novice, inexperienced surgeons.

Tremp et al. suggested a treatment algorithm based on preservation rhinoplasty (PR) through a systematic analysis of the nasal septum in crooked nose cases [15]. PR is a more conservative and much less aggressive approach that results in greater patient satisfaction [41,42]. The techniques described by the authors require an experienced surgeon.

Rohrich et al. developed an algorithm for correcting a crooked nose in 2002; however, the authors did not test its effectiveness on a series of patients in their paper [2]. In a 2017 paper, the authors focused on the association between nasal deviation and concomitant facial asymmetry. The paper offered many valuable comments on the proposed surgical techniques but did not include a new management algorithm [34].

Referring to the literature, Cheng et al. described a classification and corresponding surgical algorithm for a crooked nose in Asian patients [18]. Ethnic diversity in nasal anatomy is a crucial factor in rhinoplasty, particularly in the context of trauma, as different populations have distinct nasal features shaped by genetic, environmental, and cultural factors. These differences should be acknowledged, especially when considering how trauma may affect nasal structures. Nasal trauma can exacerbate these structural differences, potentially leading to unique challenges in the healing process and surgical correction. For example, individuals of African descent with thicker skin and a low, wide dorsum may find it more difficult to achieve a refined tip or ensure that the nasal cartilages retain their shape after surgery. In the case of non-Caucasian patients with trauma nasal deformities, grafts may be required to reconstruct the nasal dorsum, particularly in individuals with a less prominent dorsum. In the case of non-Caucasian noses, the surgeon often lacks sufficient septal cartilage for grafting due to the nasal structure characteristic of the patient’s ethnic background. Cartilage can be harvested from the ear or rib. Whereby, Pozzi et al. [7] suggest harvesting rib cartilage for Afro-descendant noses due to the need for a larger volume of appropriately thick reconstructive material in this group of patients. Therefore, it is crucial to conduct a thorough preoperative assessment and discuss with the patient the reasons for the location of cartilage graft harvesting during the initial consultation [7,43]. Cheng et al. [18] emphasized the role of septoplasty and correctly performed osteotomies. However, the nasal pyramid is not as prominent in Asians as in Caucasians, whose nasal bones are much longer. This affects the planning of the number and type of osteotomies performed. Like Pascali et al. [44], Cheng stressed that fixing a crooked nose should not just involve correcting the nasal bones and septum; special attention should be paid to the lateral nasal cartilage and soft tissues. Due to the thick skin and soft tissue envelope (SSTE) found in Asians, obstruction due to internal valve collapses is extremely rarely observed, which translates into the use of spreader grafts [18,32]. As demonstrated by Cheng et al. [18], spreader grafts are mainly used to straighten out high dorsal deviation in contrast to Caucasian patients, where they are a mainstay of treatment for insufficient internal nasal valves and are commonly placed preventively during rhinoplasty, after hump removal, to avoid middle vault collapse [37].

The study used bilateral spreader grafts in 60 patients, of which 56 were asymmetric (one of the grafts was thicker) and 29 were unilateral. Clocking sutures were used in all patients. Septal cartilage material was used to create grafts in 72 patients, and additional cartilage from the auricle was used in 17 patients. Cartilage obtained from the auricle is often used to structurally reinforce areas such as the nasal tip or middle third. Because of its flexibility, ease of obtaining, and low risk of calcification with age, which is the case with rib cartilage, it is a particularly desirable material for nasal asymmetry, refinement of the tip, or strengthening of the nasal dorsum. When planning surgery using cartilage from the patient’s auricle, the surgeon should have a thorough discussion, explain realistic expectations, obtain informed consent for the extra material to be harvested, and explain the surgical approach in simple terms. Patients often express concerns about visible scarring, a change in the shape of the auricle, or compromised function of the operated ear. The surgeon’s role is to clarify any concerns, including a thorough discussion of the location of the incision (usually the area behind the auricle), and reassure the patient of no change in the shape of the auricle and the function of the ear.

Algorithms for crooked noses aim to correct the structural deformity and the functional impairment that often accompanies it. Patients’ quality of life usually improves significantly after surgery, as both the improvement in the patient’s appearance and the function of the nose contribute to a greater sense of self-esteem and satisfaction. After statistical analysis, our study showed that the scores of all measured SCHNOS parameters (both the cosmesis domain (SCHNOS-C) and the obstruction domain (SCHNOS-O)) were significantly lower after surgery [9]. At the same time, significantly higher ROE scores were observed, indicating that patients were more satisfied with the outcomes of their plastic surgery. The results clearly and objectively indicate the benefits of the surgery performed. Comparing other papers, Won et al. [40] evaluated postoperative outcomes in 25 patients with a post-traumatic crooked nose using a telephone survey, considering both aesthetic and functional issues [40]. They found that functional outcomes were better than aesthetic outcomes. However, the authors did not use validated survey instruments for assessment. The aesthetics of the nose and nasal patency are worth highlighting here. Patients with a post-traumatic crooked nose showed greater distress in the aesthetic component (SCHNOS-C: 22.6) than in the functional component (SCHNOS-O: 11.7). After surgery, statistically significant improvements were observed in both domains (SCHNOS-C and SCHNOS-O: 3.1 and 3.0, respectively). This can be explained by a number of psychological factors, such as the effect of the nose’s appearance on self-esteem and its subsequent translation into satisfaction with the functional outcome. Changes in facial appearance, especially in the nasal area, can lead to significant emotional stress and a reduced quality of life for patients, highlighting the importance of the aesthetic aspect in post-traumatic surgery [45,46,47].

Analyzing the available literature comparing patient satisfaction after rhinoplasty in patients with and without a history of nasal trauma, some differences in the outcomes and expectations of the two groups are indicated. Patients with a history of nasal trauma often report different motivations compared to patients who undergo surgery solely for aesthetic and less functional reasons. It is not uncommon for trauma patients to prioritize the restoration of function and correction of nasal asymmetry, which often translates into greater satisfaction with achieving these goals. However, the surgeon should bear in mind that trauma-related cases may also require more complex surgical techniques due to what may, unfortunately, carry an increased likelihood of revision surgery, potentially affecting the satisfaction of some patients [36].

It is worth noting that several methods are usually available for rhinoplasty, allowing the surgeon to achieve the same or similar outcomes using different surgical techniques. The authors did not aim to compare the effectiveness of the different surgical techniques but to create a simple algorithm that is accessible to the novice surgeon to facilitate the planning and execution of the procedure in a patient with a post-traumatic crooked nose. The effectiveness of their proposed algorithm has been demonstrated through evaluation and statistical analysis of quality of life. We believe this paper will provide valuable guidance and contribute to satisfactory postoperative outcomes, especially among inexperienced surgeons.

## 6. Conclusions

Achieving satisfactory aesthetic and functional outcomes requires a comprehensive understanding of nasal anatomy, a thorough preoperative assessment, clear communication with the patient about realistic expectations, and the skill to execute a wide range of surgical techniques. During rhinoseptoplasty of a crooked nose, not only aesthetic but also functional aspects are important, as the external deformity is often closely related to impaired nasal airflow. If performed correctly, the procedure improves the patient’s quality of life. Preoperative and postoperative quality of life questionnaires (ROE, SCHNOS) revealed a significant improvement, with aesthetic aspects showing greater enhancement than functional aspects.

## Figures and Tables

**Table 1:** Surgical procedure algorithm for a crooked nose.

Surgical Procedure Algorithm For a Crooked Nose:
1. Open approach
2. Septum management
3. Nasal conchae reduction
4. Osteotomies and osteoplasty
5. Middle third of the nose restoration
6. Nasal tip suturing
7. Final camouflage
8. Dressing

**Table 2:** Mean questionnaire results ROE and SCHNOS.

	Group	Mean (±SD)	Median
Preoperative	ROE	8.7 (±4.4)	8
	SCHNOS	46.0 (±14.6)	46
	SCHNOS-O	11.7 (±5.8)	12
	SCHNOS-C	22.6 (±6.4)	24
Postoperative	ROE	20.2 (±2.7)	21
	SCHNOS	9.1 (±8.6)	6
	SCHNOS-O	3.0 (±2.7)	2
	SCHNOS-C	3.1 (±4.9)	2

**Table 3:** Results of the normal distribution assessment in the study groups using the Kolmogorov–Smirnov test.

	Group	*p*-Value
Preoperative	ROE	>0.05
	SCHNOS	>0.05
	SCHNOS-O	>0.05
	SCHNOS-C	<0.05
Postoperative	ROE	<0.05
	SCHNOS	<0.05
	SCHNOS-O	<0.05
	SCHNOS-C	<0.05

**Table 4:** Assessment of the significance of differences between SCHNOS and ROE prior and after surgery.

	Mean Preoperative (±SD)	Mean Postoperative (±SD)	U	*p*-Value
ROE	8.7 (±4.4)	20.2 (±4.9)	181	<0.001
SCHNOS	46.0 (±14.6)	9.1 (±8.6)	148.5	<0.001
SCHNOS-O	11.7 (±5.8)	3.0 (±2.7)	836.5	<0.001
SCHNOS-C	22.6 (±6.4)	3.1 (±4.9)	275.5	<0.001

## Data Availability

Medical data of enrolled patients is present in the Department of Otolaryngology with Division of Cranio-Maxillo-Facial Surgery.
